# RNA-Seq Analysis Provides the First Insights into the Phylogenetic Relationship and Interspecific Variation between *Agropyron cristatum* and Wheat

**DOI:** 10.3389/fpls.2017.01644

**Published:** 2017-09-21

**Authors:** Shenghui Zhou, Baiqiang Yan, Fei Li, Jinpeng Zhang, Jing Zhang, Huihui Ma, Weihua Liu, Yuqing Lu, Xinming Yang, Xiuquan Li, Xu Liu, Lihui Li

**Affiliations:** National Key Facility for Crop Gene Resources and Genetic Improvement, Institute of Crop Science, Chinese Academy of Agricultural Sciences Beijing, China

**Keywords:** wheat, wild relatives, phylogenetic relationship, interspecific variation, SNP, RNA-Seq

## Abstract

*Agropyron cristatum*, which is a wild grass of the tribe Triticeae, grows widely in harsh environments and provides many desirable genetic resources for wheat improvement. However, unclear interspecific phylogeny and genome-wide variation has limited the utilization of *A. cristatum* in the production of superior wheat varieties. In this study, by sequencing the transcriptome of the representative tetraploid *A. cristatum* Z559 and the common wheat variety Fukuhokomugi (Fukuho), which are often used as parents in a wide cross, their phylogenetic relationship and interspecific variation were dissected. First, 214,854 transcript sequences were assembled, and 3,457 orthologous genes related to traits of interest were identified in *A. cristatum*. Second, a total of 72 putative orthologous gene clusters were used to construct phylogenetic relationships among *A. cristatum*, Triticeae and other genomes. A clear division between *A. cristatum* and the other Triticeae species was revealed. Third, the sequence similarity of most genes related to traits of interest is greater than 95% between *A. cristatum* and wheat. Therefore, using the 5% mismatch parameter for *A. cristatum*, we mapped the transcriptome sequencing data to wheat reference sequences to discover the variations between *A. cristatum* and wheat and 862,340 high-quality variants were identified. Additionally, compared with the wheat A and B genomes, the P and D genomes displayed an obviously larger variant density and a longer evolutionary distance, suggesting that *A. cristatum* is more distantly related to the wheat D genome. Finally, by using Kompetitive Allele Specific PCR array (KASPar) technology, 37 of 53 (69.8%) SNPs were shown to be genuine in Z559, Fukuho, and additional lines with seven different P chromosomes, and function of the genes in which these SNPs are located were also determined. This study provides not only the first insights into the phylogenetic relationships between the P genome and Triticeae but also genetic resources for gene discovery and specific marker development in *A. cristatum*, and this information will be vital for future wheat-breeding efforts. The sequence data have been deposited in the Sequence Read Archive (SRA) database at the NCBI under accession number SRP090613.

## Introduction

Wheat (*Triticum aestivum* L., 2*n* = 6x = 42, genomes AABBDD) is one of the most important crops. Improving the productivity, adaptation, quality, and nutritional value of wheat to meet the demand for cereal crops remains a major challenge in modern agriculture. However, the narrow genetic basis of wheat has become a bottleneck for wheat improvement, which has led to a surge of interest in exploring natural biodiversity as a source of novel alleles to deliver high-performing wheat varieties (Dubcovsky and Dvorak, 2007). Wild wheat relatives harbor superior agronomic traits, and hybridization is considered an effective measure to enrich the genetic base that has been widely used in wheat-breeding programmes (Bevan et al., 2017).

**Figure 1:**
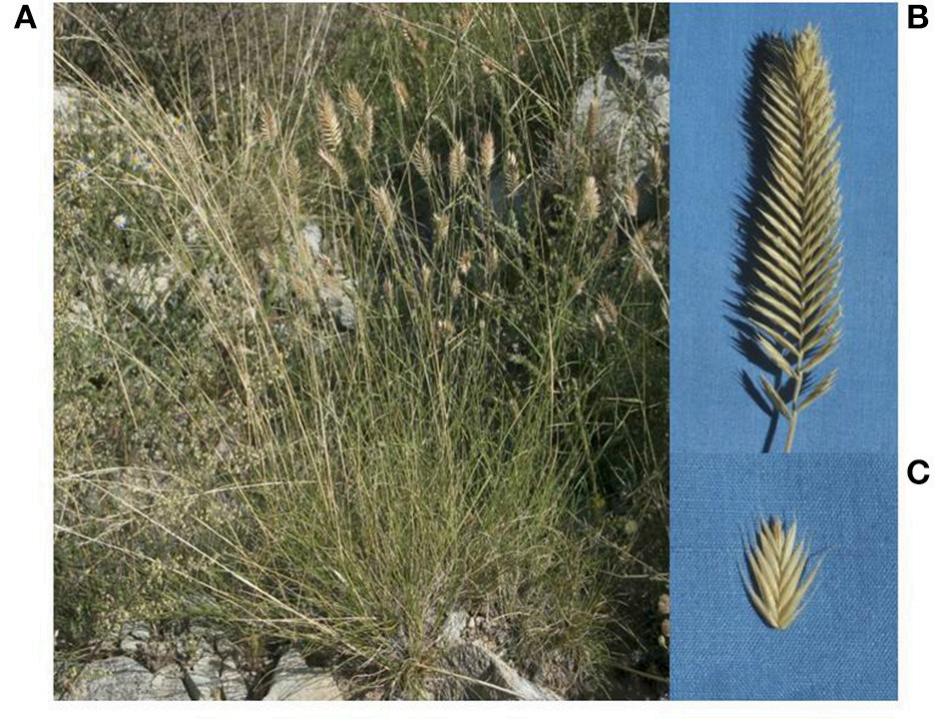
Morphological characterization of *A. cristatum*. **(A)** The *A. cristatum* growing in the natural environment. **(B)** Spike morphological characterization. **(C)** Spikelet morphological characterization.

*Agropyron* is a perennial genus of the tribe Triticeae, which is commonly called the crested wheatgrass complex, and is built upon one basic P genome, including 3 ploidy levels, diploid, tetraploid, and hexaploid (Dewey, 1984). Most *Agropyron* species, as excellent sources of forage, have mainly been used as forage crops with high economic value and have also served as a tertiary gene pool for wheat improvement (Dewey, 1984). The tetraploid crested wheatgrass *Agropyron cristatum* (L.) Gaertn. (2*n* = 4x = 28; genomes PPPP) is the most common member of the *Agropyron* genus. *A. cristatum* is native to Europe and Asia, especially in low-temperature grasslands and sands regions in Eurasian, and has short broad spikes that taper at the top, small seeds, and short stature (Chen et al., 2013) (Figure 1). *A. cristatum* not only provides protein as a forage source but also possesses several desirable traits for wheat improvement, such as more tillers, spikelets, and florets, resistance against powdery mildew, barley yellow dwarf virus, leaf rust, stripe rust, and stem rust and tolerance against salinity, drought, and low temperatures. In the early 1990s, the inter-generic hybridization of wheat cv. Fukuhokomugi (Fukuho) and *A. cristatum* accession Z559 was successfully completed via a wide cross and embryo rescue (Li, 1995), and then, many additional lines with excellent characteristics, including disomic substitution lines, translocation lines, and introgression lines, were produced (Zhang et al., 2015b; Li et al., 2016). Several of these lines have been used in wheat-breeding programmes, such as Pubing3504 and Pubing3228, which exhibit an increased number of spikelets and florets (Chen et al., 2012). Although their growth characteristics and utilization in wheat-breeding programmes have been extensively investigated, little is known regarding the phylogenetic relationship and genetic diversity between *A. cristatum* and wheat.

Molecular markers are important not only for studies regarding evolutionary processes, novel functional gene discovery and development of conservation strategies but also for effectively using genetic resources in breeding programmes. In total, 34 polymorphic markers in *A. cristatum* and wheat have been identified from 48 *A. cristatum*-specific sequences obtained from degenerate oligonucleotide-primed PCR (DOP-PCR) products (Han et al., 2017). Additionally, Wu et al. (2010) developed three sequence-characterized amplified region (SCAR) markers that were specific for the P genome of *A. cristatum*. However, attempts to understand the genetic diversity and phylogenetic relationships between Z559 and Fukuho using these molecular markers have been limited. The identification of single-nucleotide polymorphisms (SNPs) via next-generation sequencing (NGS) is efficient and cost-effective. In addition, because SNPs are biallelic and are the most abundant genetic variations, SNP are evenly distributed at higher frequencies throughout the genome of most plant species (Allen et al., 2011). Therefore, SNP markers have become increasingly useful for developing high-density genetic maps, conducting genome-wide association mapping, marker-assisted selection (MAS) and genomic selection (GS) studies and assessing genetic diversity. Winfield et al. (2016) used a wheat NimbleGen array (Winfield et al., 2012) to direct the capture and targeted re-sequencing of the wheat exome and identified a large number of SNPs from 43 bread wheat accessions and wheat relatives. In addition, 218 genome-wide wheat/*Ambylopyrum muticum* introgressions were detected and characterized using these SNP markers and the Axiom genotyping array (King et al., 2017).

NGS has facilitated the large-scale discovery of SNPs in various model and non-model plant species. SNP discovery from NGS data was also combined with bulked segregant analysis for the fine mapping of genes in polyploid wheat (Trick et al., 2012; Ramirez-Gonzalez et al., 2015; Bassi et al., 2016). RNA-sequencing (RNA-Seq) has also recently become a popular technique because it is cost-effective, does not rely on a reference genome and can contribute to transcriptional analysis, gene discovery, phylogenetic analysis, and molecular marker development (Meena et al., 2016). In a previous study, *de novo* transcriptome assembly and unigene functional annotation were conducted in *A. cristatum*, and gene resources related to stress resistance and inflorescence development and specific to *A. cristatum* within the tribe Triticeae were identified (Zhang et al., 2015a). However, the phylogenetic relationships, genetic diversity and development of SNP molecular markers in *A. cristatum* and wheat, especially in inter-generic hybrid parents used in our lab (wheat cv. Fukuho and *A. cristatum* accession Z559), have been poorly investigated.

**Figure 2:**
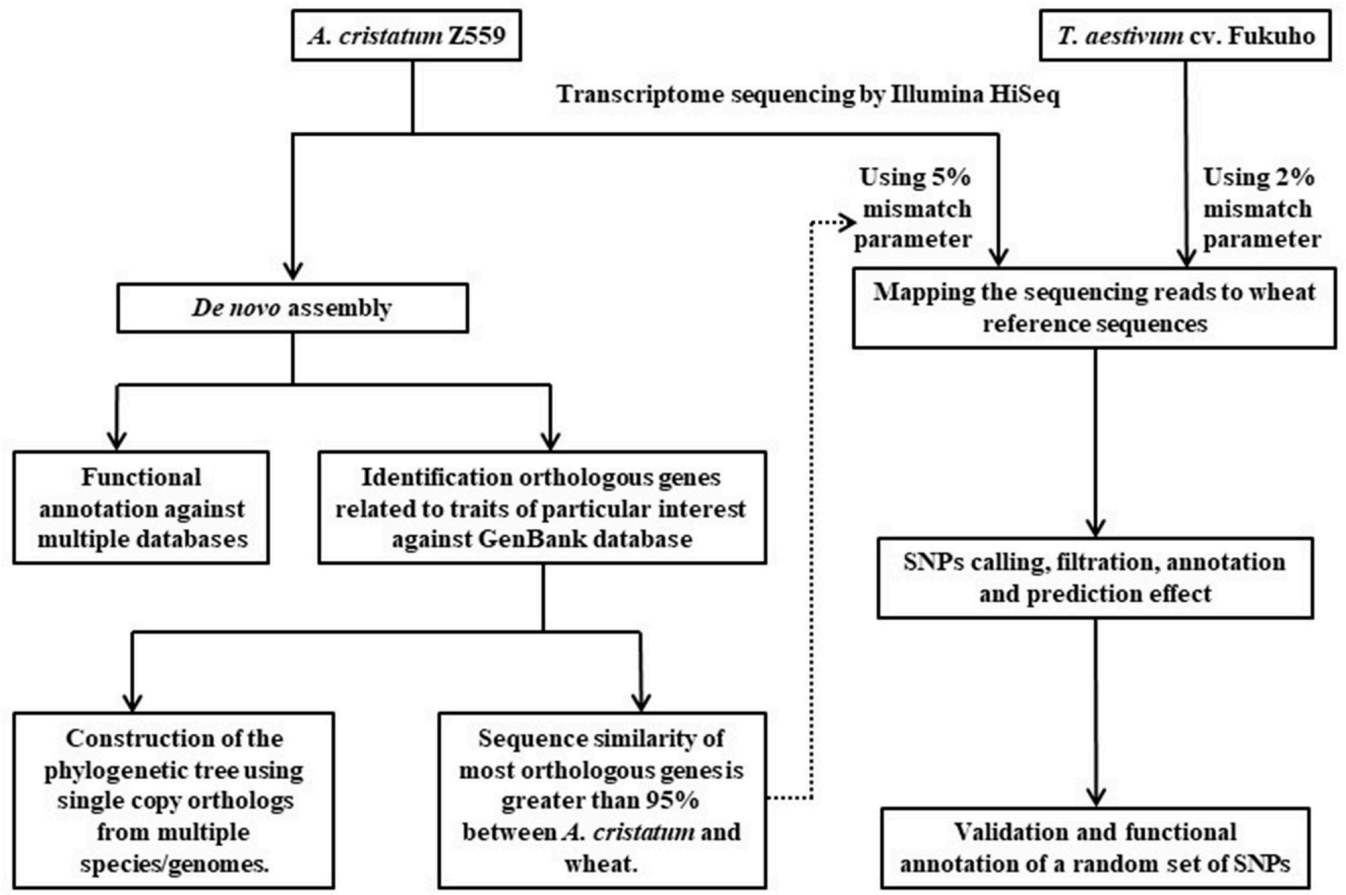
Pipeline used to dissect phylogenetic relationship and interspecific variation between *A. cristatum* and wheat in this study.

In this study, a pipeline was used to dissect phylogenetic relationship and interspecific variation between *A. cristatum* and wheat (Figure 2). Transcriptome sequencing was performed using Illumina HiSeq 2500 in Z559 and Fukuho. Sequencing data of Z559 were assembled and annotated, and candidate genes related to traits of interest were identified. Furthermore, to better understand the evolutionary history of *A. cristanum* (P genome), a phylogenetic tree for *A. cristatum*, Triticeae, and some other genomes was constructed using single-copy orthologous genes. Then, according to the sequence similarity, the trimmed sequencing data were mapped against the wheat reference genome to discover and characterize variants, reveal the genetic diversity at the transcriptome level, and create large subsets of markers for *A. cristatum* and wheat.

## Materials and methods

### Plant materials

The *A. cristatum* accession Z559 (2*n* = 4x = 28, PPPP, from Xinjiang, China) has been previously described (Zhang et al., 2015a). *T. aestivum* cv. Fukuho was used as a recipient parent for the wide cross between wheat and *A. cristatum* (Li, 1995).

### RNA extraction and sequencing

The seedlings of *A. cristatum* Z559 were planted in the greenhouse. Then, *A. cristatum* Z559 using tillers planting and Fukuho were cultivated in the same experimental field of the Chinese Academy of Agricultural Sciences (E116.33, N39.96), Beijing, China. The type of soil was sandy clay loam. Sowing date was October 1 of 2015 and experimental field was fertilized with 112 kg ha^−1^ of a N-P-K mixture (16–20–14%) plus 170 kg ha^−1^ of urea. Plants were watered as needed. Unfertilized caryopses and young healthy leaves were collected from five different plants at growth stage 54 (Zadoks et al., 1974) and immediately snap-frozen in liquid nitrogen, ground into powder, and then extracted using TRIzol Reagent (Invitrogen, Carlsbad, CA, USA) according to the manufacturer's recommendations. The quantity and integrity of the total RNA were assessed using an Agilent 2100 Bioanalyzer (Agilent Technologies, Palo Alto, CA, USA) and 1% agarose gel electrophoresis. Equal amounts of total RNA from each tissue were pooled for Z559 and Fukuho. Only the total RNA samples with RIN values ≥8 were used for constructing the cDNA libraries. All libraries were sequenced using the Illumina HiSeq 2500 platform with a paired-end read length of 150 bp. The library construction and sequencing were performed by the Novogene Corporation (Beijing, China).

### Data pre-processing, *de novo* assembly, and annotation

The raw sequence reads were cleaned by removing the RNA adapters and trimming the low-quality bases (Q < 20) with a minimum read length of 36 bases using Trimmomatic (version 0.36) (Bolger et al., 2014). The trimmed reads were then *de novo* assembled using the Trinity package (version 2.4.0) with the default parameters and a minimum transcript length setting of 200 nt (Haas et al., 2013). The clustering of the transcripts was performed using the CD-HIT-EST software (version 4.6.6) (Li and Godzik, 2006) to reduce the unavoidable redundancy produced by the Trinity assembly. Many low-quality transcripts and potential assembly errors can occur during assembly. Therefore, all reads were mapped back to transcripts using Bowtie (version 1.2.0) (Langmead et al., 2009). The fragments per kilobase of transcript per million mapped reads (FPKM) value of the transcripts was calculated using RSEM (version 1.3.0) (Li and Dewey, 2011), and transcripts with FPKM values <1 were also removed. The remaining transcripts were then used to perform the downstream analysis.

The filtered transcripts were analyzed by TransDecoder (version v3.0.0) (Grabherr et al., 2011) to determine their cDNA coding sequence (CDS) and protein sequences. For the annotation, all CDSs were aligned using BLAST (version 2.5.0+) (Altschul et al., 1990) to the Non-Redundant (NR) database, Pfam-A database (version 31.0) (Finn et al., 2014), SwissProt database (release 2017_04), and Eukaryotic Orthologous Groups (KOG) database (with E-value cut-off of 1e-5) (Tatusov et al., 2003). The protein sequence with the best hit was considered the optimal annotation. The results were loaded into Trinotate (version 3.0) (Grabherr et al., 2011) SQLite database to generate a comprehensive annotation report.

### Ortholog identification and phylogenetic analysis

Genes potentially related to traits of particular interest were searched against GenBank sequence database (http://www.ncbi.nlm.nih.gov/genbank/) using an in-house Perl script. All traits of particular interest were more concerned in wheat breeding programmes and classified into three major categories. These categories were as follows: (i) agronomic traits, which included kernels per spike, seed weight, tiller, plant height, photosynthetic efficiency, stem toughness, and growth period-related traits; (ii) biotic stress-related traits, which included fungal/virus disease and insect pests resistance-related traits; and (iii) abiotic stress-related traits, which included cold, heat, drought and salt stress, fertilizer and water use efficiency, seed storage and adaptation-related traits (Tables S1, S2 and Files S1, S2). The clustering of genes was performed using the CD-HIT-EST software to obtain the representative genes. OrthoMCL (version 2.0.9) was used to identify the orthologous genes in *A. cristatum* (Li L. et al., 2003). A sequence similarity analysis was also performed using BLAST with the default parameters to determine the sequence identity.

The agronomic traits and biotic and abiotic stress-related genes identified above in *A. cristatum* were used to find orthologous genes in other genomes, e.g., *Triticum aestivum* (A, B, and D genomes were separated), *Triticum urartu, Triticum turgidum* (A and B genomes were separated), *Aegilops tauschii, A. tauschii, Secale cereale, Brachypodium distachyon, Sorghum bicolor, Zea mays, Setaria italica, Oryza sativa*, and *Arabidopsis thaliana*. The cDNA sequences of these genomes were downloaded from EnsemblPlants (www.plants.ensembl.org), and OrthoMCL was used to identify orthologous gene pairs. Putative orthologs with a BLAST score ratio of the second best-hit to the first best-hit greater than 0.8 were discarded, which can yield single-copy genes in one genome. Gene clusters that are single copy in one genome and highly conserved in all genomes were aligned by MUSCLE (Edgar, 2004). The alignment was subjected to MEGA7 (Kumar et al., 2016) to construct the phylogenetic tree using the Neighbour-Joining method (Saitou and Nei, 1987). The bootstrap consensus tree inferred from 500 replicates is taken to represent the evolutionary history of the analyzed taxa (Felsenstein, 1985). Branches corresponding to partitions reproduced in less than 50% of bootstrap replicates were collapsed. Evolutionary distances were computed using the Maximum Composite Likelihood method (Tamura et al., 2004) and are in units of the number of base substitutions per site. The tree is drawn to scale, with branch lengths in the same units as those of the evolutionary distances used to infer the phylogenetic tree. All positions containing gaps and missing data were eliminated.

### SNP discovery in the transcriptomes of *A. cristatum* and wheat

The raw reads were aligned to the wheat reference genome sequence TGACv1 (Clavijo et al., 2017) using the STAR tool (version 2.5.3a) (Dobin et al., 2013), including the 2-pass STAR method with a minimum intron length of 20 bp, a maximum intron length of 20 kb the default settings for the other parameters and 5 and 2% maximum mismatches for *A. cristatum* and wheat, respectively. The creation of the GTF annotation file and the quantification of the genes and isoforms were performed using StringTie (version 1.3.3b) (Pertea et al., 2015) from a mapping bam file. Duplicate markings, split “N” Trimming and reassigning mapping qualities were successively performed to filter the mapping results. The haplotype caller in the GATK variant pipeline was used to call SNPs between *A. cristatum* and wheat from the genome-mapped alignments. The variant filtration was performed with a total coverage >6 and an absolute value of allele frequency difference >0.9 between *A. cristatum* and wheat to obtain high-quality SNPs. The SnpEff (version 4.3k) pipeline was used to annotate and predict the effects of the SNPs based on their genomic locations and predict their coding effects (Cingolani et al., 2012), providing a simple assessment of the putative impact of the variant, i.e., high (frame shifts, addition/deletion of stop codons, etc.), moderate (codon change/deletion/insertion, etc.), low (synonymous changes, etc.), and modifier (changes outside coding regions, etc.). The definitions of the putative impact of the variants are listed in the Table S3 with brief explanations. The visualization of the data at the genome-wide level (TGACv1 map) was performed using the software Circos (version 0.69-3) (Krzywinski et al., 2009).

### SNP marker validation

Young leaves of Z559, Fukuho and their additional lines involving seven different P chromosomes were used for DNA extraction, PCR amplification, and SNP marker validation. In total, 53 SNP markers were validated using the Kompetitive Allele Specific PCR (KASP) array. The SNP contextual sequences that did not cross the intron in the wheat genome were obtained and used to design the primers. Based on the 53 SNP loci contextual sequence, two allele-specific primers (one for each SNP allele) and one common (reverse) primer were designed for each KASP assay using a tool provided by LGC Genomics (www.lgcgenomics.com) (Table S4). The KASP assays were designed by LGC Genomics and carried out according to the company's protocol (http://lgcgenomics.com).

## Results

### Sample sequencing

Two transcriptome libraries were generated from the pooled RNA-extracts from two tissues (leaves and young spikes) of Z559 and Fukuho. The sequencing produced paired-end reads with a length of 150 base pairs (bp). A summary of the transcriptome sequencing data is presented in Table 1. The Illumina sequencing generated 64,254,354 and 42,700,222 sequence reads in Z559 and Fukuho, respectively. After filtering the low-quality reads, 99.98% of the sequencing reads (64,243,343 reads for Z559 and 42,692,045 reads for Fukuho) were retained, and a downstream analysis was performed. GC content (52%) in the sequences of Z559 and Fukuho was identical (Table 1). The sequence data have been deposited in the Sequence Read Archive (SRA) database at the NCBI under the accession number SRP090613.

**Table 1:** Summary of the RNA-Seq data.

**Sample**	**Number of reads**	**Number of reads after trimming**	**After trimming data/raw data (ratio) (%)**	**Average GC content (%)**
Z559	64,254,354	64,243,343	99.98	52.00
Fukuho	42,700,222	42,692,045	99.98	52.00

### *A. cristatum* Z559 transcriptome assembly and gene annotation

**Figure 3:**
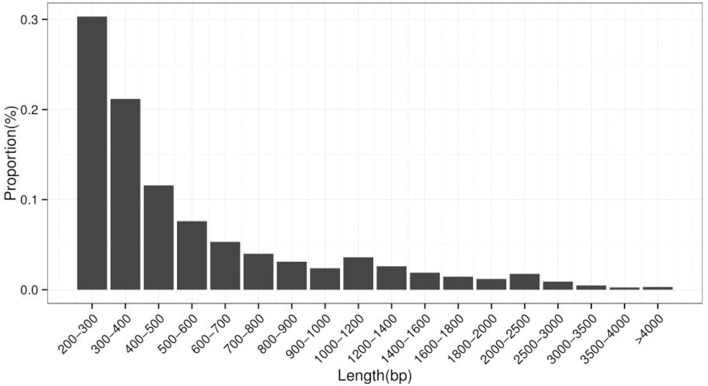
Transcript length distribution in *A. cristatum*.

A *de novo* assembly of Z559 was performed using the high-quality filtered reads. The assembly produced a total of 214,854 transcripts ranging in length from 201 to 13,610 bp with a mean size of 611.22 bp, and the size of N50 was 792 bp (Table 2, Figure 3).

**Table 2:** Statistics of the *A. cristatum* transcriptome assembly and annotation.

**Assembly/annotation parameters**	
Number of transcripts assembled	214,854
Maximum transcript length (bp)	13,610
Minimum transcript length (bp)	201
Average transcript length (bp)	611
N50 length (bp)	792
Number of transcripts with coding sequences	78,912
Number of transcripts with blast hits to NR	57,183
Number of transcripts with blast hits to Pfam	38,208
Number of transcripts with blast hits to SwissProt	55,157
Number of transcripts with blast hits to KOG	43,487
Number of transcripts with GO terms	18,073
Number of transcripts with at least one hit in these databases	66,346

Of the assembled transcripts, 78,912 (36.7%) transcripts with coding sequences were identified using TransDecoder software (Grabherr et al., 2011). Of the predicted transcripts, 57,183 transcripts were found to have homologs in the NR database. The number of transcripts with Pfam domain assignments was 38,208, and 55,157 transcripts had significant matches in the SwissProt database. In addition, 18,073 transcripts were associated with GO terms, and 43,487 transcripts had matches in the KOG database. Altogether, 66,346 transcripts had at least one hit in these databases (Table 2).

### Identification of orthologous genes and phylogenetic analysis

To further understand and better use the genetic diversity of *A. cristatum*, genes potentially related to agronomic and biotic and abiotic stress-related traits were identified in *A. cristatum*. In total, 9,354 genes related to these traits were downloaded from GenBank (Table S1). After filtering the highly homologous genes, 4,841 representative genes remained (Tables S1, S2 and Files S1, S2). Furthermore, 3,457 genes orthologous to these representative genes were identified in our assembled Z559 transcripts (Table S1).

**Figure 4:**
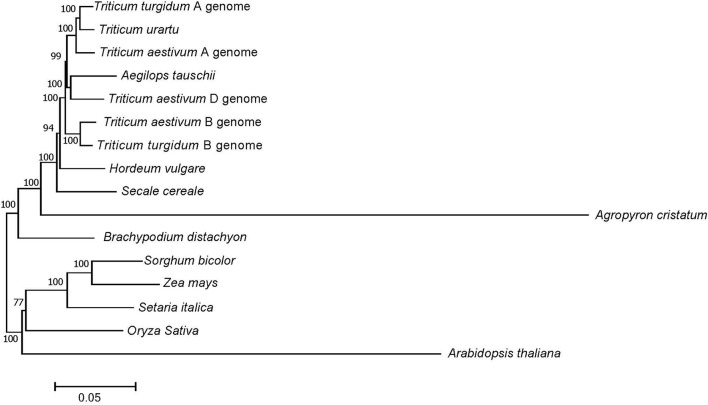
Phylogenetic relationships between *A. cristatum* and other genomes. Numbers at each node are the bootstrap values, shown as percentages. The branch lengths are the same as the evolutionary distances.

Of the 3,457 genes identified above, a total of 72 putative orthologous gene clusters were identified after carefully filtering with one copy in one genome and conversed in all genomes. Phylogenetic relationships were then constructed based on the alignment of these 72 orthologs from *A. cristatum* transcriptome data and cDNA data from other genomes (Figure 4). Specifically, *A. cristatum* and other Triticeae (Poaceae), including *T. aestivum* and their ancestral species *T. urartu, A. tauschii* and *T. turgidum, Hordeum vulgare*, and *S. cereale*, clustered into a sister clade. However, four major clades existed within Triticeae, and the analysis also showed a clear division between *A. cristatum* and the other Triticeae species, indicating the P genome should possess rich genetic variation that can be used for wheat genetic improvement. The results also supported the point that hybridization between *Agropyron Gaertn*. and wheat is difficult to achieve (Dewey, 1984). Moreover, the evolutionary distance indicated that the P genome was more distantly related to the wheat D genome than to the A and B genomes (Figure 4).

### Variant calling and effects between *A. cristatum* and *T. aestivum*

**Figure 5:**
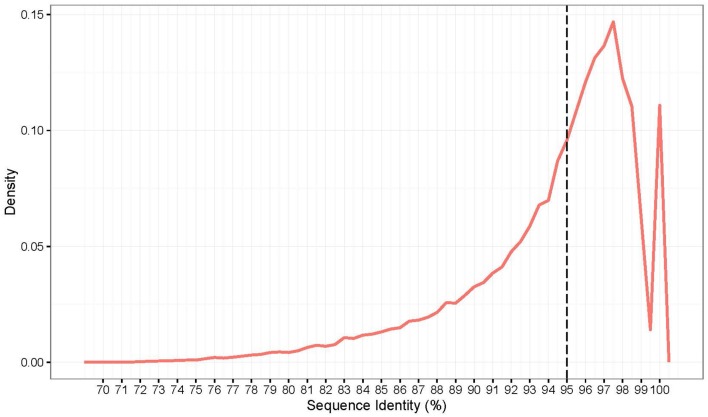
Density distribution of *A. cristatum* transcript sequences identity compared to the wheat.

In this study, *A. cristatum* transcript sequences assembled using Trinity were compared with the published wheat CDS database using a blastn search. Sequence identity averages of 94.2% were found between the wheat and *A. cristatum* transcript sequences (Figure 5). The highest peak of the distribution indicates a 97.6% identity between the *A. cristatum* transcriptome and the CDS of wheat (Figure 5). Overall, the transcript sequences of *A. cristatum* show highly conserved wheat transcript sequences, and most of the transcript sequences (72.5%) (Figure 5) and genes related traits of interest (83.3%) have ≥95% sequence identity to the CDS of wheat. Thus, the *A. cristatum* transcriptome is closely related to the wheat transcriptome.

**Figure 6:**
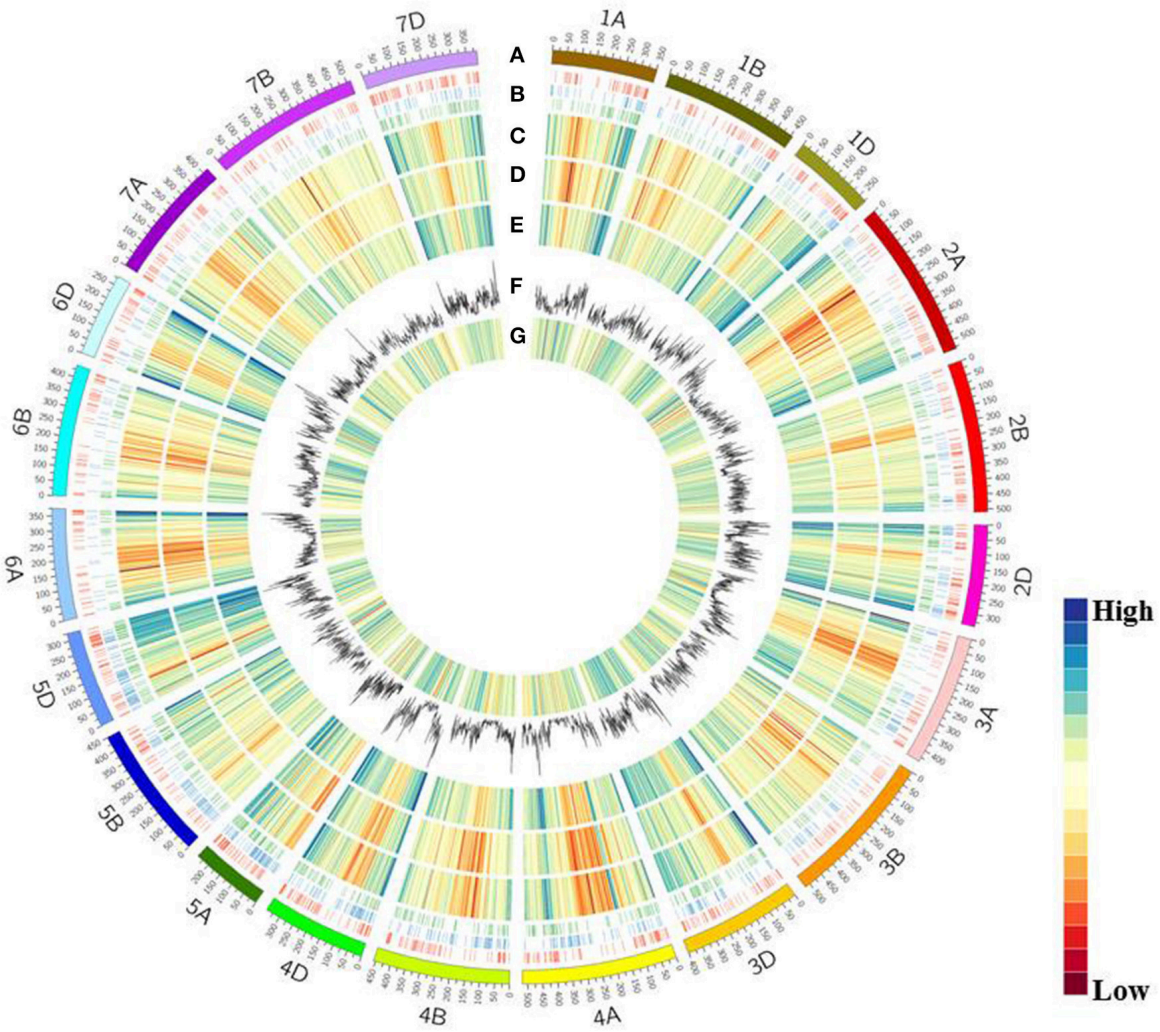
CIRCOS visualization of different data at the wheat genome-wide level. **(A)** Karyotype of the wheat genome. **(B)** Location of homologous genes, including SNPs related to traits. Agronomic traits (red), biotic stress (blue) abiotic stress (green). **(C)** Gene content density distribution; Gene density was calculated in a 3-Mb window. **(D)** Transcriptional density distribution in wheat. Transcript regions density was calculated in 3-Mb window intervals. **(E)** Transcriptional density distribution in *A. cristatum*. Transcript region density was calculated in 3-Mb window intervals. **(F)** Variant distribution by chromosome. SNP density was calculated in 3-Mb window intervals. **(G)** Variant density in transcript regions. Variant density was calculated in transcript regions at 3-Mb window intervals.

To identify local genomic variability, the Z559 and Fukuho transcriptome sequencing data were mapped to a wheat reference sequence (Figure 6A). The average gene density and transcriptional density were calculated along all chromosomes in the wheat genome. In total, 268,200 and 184,423 transcriptional regions were identified in Z559 and Fukuho, respectively. In addition, 112,765 protein-coding genes were predicted with functional support and the entire gene structure. The average gene density and transcriptional density sharply decreased from centromeres to telomeres in the whole chromosomal region in both Z559 and Fukuho (Figures 6C–E).

**Figure 7:**
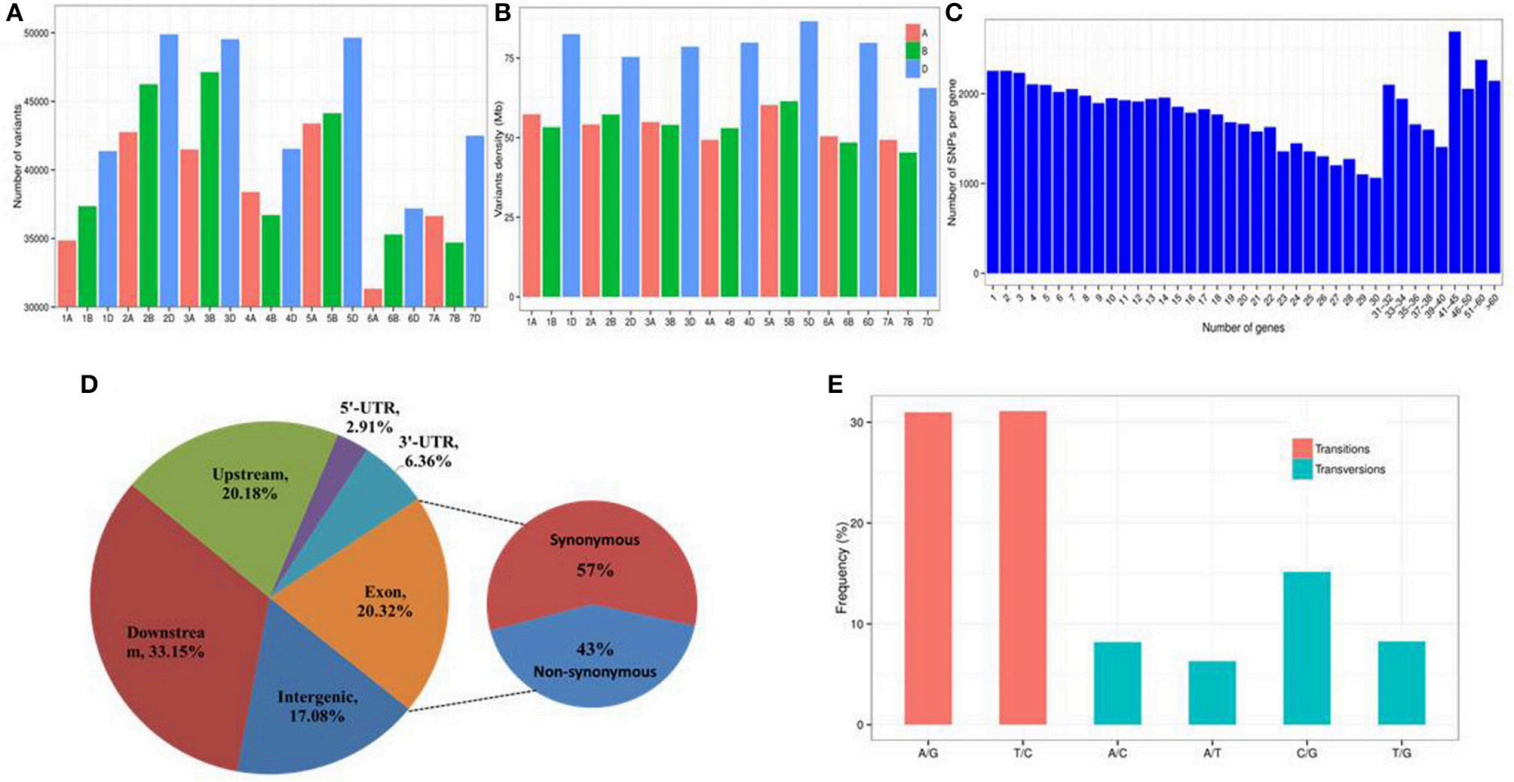
Characterization of SNPs between *A. cristatum* and wheat. **(A)** Distribution of SNPs in the wheat genome. **(B)** Density distribution of SNPs in the wheat genome. Density was measured by averaging the number of SNPs per 1 Mb of the wheat genomic region. **(C)** Distribution of the number of SNPs per gene. **(D)** Frequency of different substitution types in the identified SNPs. **(E)** Distribution of SNPs in different genomic regions.

A total of 862,340 high-quality variants were identified, including 817,970 SNPs and 44,370 InDels, in the transcripts of Fukuho and Z559, which were spread across the wheat genome (Figures 6F, 7A). The number of variants in each chromosome was not directly proportional to the chromosome length and gene number. Homologous group 2 chromosomes contained the most variants, whereas homologous group 6 chromosomes contained the fewest variants (Figure 7A). The density of the SNPs per 1 Mb of the genetic region on each chromosome is shown in Figure 7B. Overall, 59.28 SNPs/Mb were found on average across the wheat genome. Importantly, in each homologous group, the highest SNP density was observed on the D genome (77.88 SNPs/Mb), followed by the B genome (53.26 SNPs/Mb), and A genome (53.59 SNPs/Mb) (Figure 7B). Interestingly, the number of variants in the telomeric regions was the highest in the whole chromosome (Figure 6F), but the variant density in the transcriptional regions was relatively lower in the telomeric and centromeric regions than that in the other chromosomal regions (Figure 6G).

Overall, 70,456 annotated genes (62.48%) contained one or more variants (Figure 7C). Of these genes, 5,036 genes had high impact variants, 42,415 genes had low impact variants, 37,902 genes had moderate impact variants and 65,967 genes had modifier variants. The average number of SNPs per gene was 21.8, while 44.4% of the genes had fewer than 20 SNPs. Interestingly, 2,143 genes harbored more than 60 SNPs (Figure 7C), which implied that these genes exhibited high diversity. Thus, these 2,143 genes might be particularly amenable to artificial selection and are helpful for understanding the genetic diversity of *Triticeae*. The distribution of the discovered variants within various genomic features is shown in Figure 7D and Table S5. The number of variants in the intergenic, downstream, and upstream regions of the genes is 376,552 (17.08%), 730,523 (33.15%), and 444,836 (20.18%), respectively.

Approximately 204,285 (9.27%) SNPs located in the 5′- or 3′-UTR regions were identified, and 20.32% of the variants were present in coding regions in which the ratio of non-synonymous to synonymous variants was ~0.75 (163,226/220,482) (Figure 7D, Table S5), suggesting that the transcriptional regions have been under purifying selection.

The frequency of substitution transitions (62.09%; A/G: 30.99% and T/C: 31.10%) was much higher than the frequency of transversions (37.91%; A/C: 8.19%, A/T: 6.30%, C/G: 15.16, and T/G: 8.26%) (Figure 7E). The proportion of A/G transitions (30.99%) was similar to that of T/C transitions (31.10%). The frequency of each type of transversion was ~7%, except for the C/G transitions, which had a frequency of 15.16% (Figure 7E). The ratio of transitions to transversions (ti/tv) was 1.64:1.

### Validation of SNPs using the KASP genotyping assay

Fifty-three SNPs between Z559 and Fukuho identified in this study were validated using a KASP genotyping assay, in which an allele-specific primer was used to discriminate between single-nucleotide substitutions (Table S4). Thirty-seven (69.8%) of the 53 SNP loci were successfully confirmed in Z559 and Fukuho and additional lines with 7 different P chromosomes. Among these, 6, 3, 5, 1, 8, and 8 SNPs were located on the 1P, 2P, 3P, 4P, 5P, and 7P chromosomes, respectively. In addition, 1 SNP located on both 2P and 5P, 2 SNPs located on both 5P and 6P, 1 SNP located on 6P and 7P, and 1 SNP located on 5P, 6P and 7P were also determined, and 1 SNP was undetermined (Table S4). The functional information of genes in which SNPs are located was shown in Table 3 and these KASP markers could be used to follow specific genes of interest.

**Table 3:** Functional information of genes in which SNPs are located.

**SNP**	**LOCUS**	**Encoded protein**	**Related trait/pathway**
*SNP-1*	PSBD_ARATH	Photosystem II D2 protein	Photosynthesis (Armbruster et al., 2010)
*SNP-2*	PSBD_ARATH	Photosystem II D2 protein	Photosynthesis (Armbruster et al., 2010)
*SNP-3*	PSBD_ARATH	Photosystem II D2 protein	Photosynthesis (Armbruster et al., 2010)
*SNP-4*	PSBD_ARATH	Photosystem II D2 protein	Photosynthesis (Armbruster et al., 2010)
*SNP-5*	CBH32509	Trehalose synthase	Plant architecture (Chary et al., 2008)
*SNP-7*	CBH32509	Trehalose synthase	Plant architecture (Chary et al., 2008)
*SNP-8*	SPSA4_ORYSJ	Sucrose phosphate synthase 4F	Sucrose synthesis (Lutfiyya et al., 2007)
*SNP-9*	GIGAN_ORYSJ	Protein GIGANTEA	Photoperiodic flowering (Fowler et al., 1999)
*SNP-11*	COBL3_ORYSJ	Protein BRITTLE CULM1-like 4	Mechanical properties of plants (Li Y. et al., 2003)
*SNP-13*	AAT1_ARATH	Transaminase A	Nitrogen assimilation (Schultz et al., 1998)
*SNP-15*	CSLD2_ORYSI	Cellulose synthase-like protein D2	Cellulose synthase (Hazen et al., 2002)
*SNP-16*	GUN7_ORYSJ	Endoglucanase 7	Internode elongation (Zhou et al., 2006)
*SNP-17*	BAI52979	G1-like1 protein	Floral organs development (Yoshida et al., 2009)
*SNP-20*	HDA19_ARATH	Histone deacetylase 19	Pathogen response (Zhou et al., 2005)
*SNP-21*	GH38_ORYSI	Auxin-responsive GH3-like protein 8	Floral organs development (Prasad et al., 2005)
*SNP-22*	GH38_ORYSI	Auxin-responsive GH3-like protein 8	Floral organs development (Prasad et al., 2005)
*SNP-28*	UGDH4_ORYSJ	UDP-glucose 6-dehydrogenase 4	Disease resistance (Klinghammer and Tenhaken, 2007)
*SNP-32*	SGT1_ORYSJ	Protein SGT1 homolog	Disease resistance (Wang et al., 2008)
*SNP-33*	GDT13_ORYSJ	GDT1-like protein 3	Disease resistance (Rice Chromosomes 11 and 12 Sequencing Consortia, 2005)
*SNP-34*	AKP45150	Zinc finger protein	Disease resistance (Hurni et al., 2015)
*SNP-35*	VPS4_ARATH	Protein SUPPRESSOR OF K(+) TRANSPORT GROWTH DEFECT 1	Plant defense (Wang et al., 2014)
*SNP-37*	ALN98169	RING finger ubiquitin E3 ligase	Heat tolerance (Liu et al., 2016)
*SNP-38*	FENR2_ORYSJ	Ferredoxin–NADP reductase	Photosynthesis (Aoki et al., 1995)
*SNP-39*	MCCA_ORYSJ	MCCase subunit alpha	Leaf senescence (Lee et al., 2001)
*SNP-40*	AAO72583	PHD-finger protein	Stress response (Cooper et al., 2003)
*SNP-41*	RENT1_ARATH	ATP-dependent helicase UPF1	Plant defense (Jeong et al., 2011)
*SNP-42*	RENT1_ARATH	ATP-dependent helicase UPF1	Plant defense (Jeong et al., 2011)
*SNP-44*	C3H41_ORYSJ	E3 ubiquitin-protein ligase makorin	Germinating (Arumugam et al., 2007)
*SNP-45*	MPK4_ARATH	Mitogen-activated protein kinase 4	Abiotic stresses (Ichimura et al., 2000)
*SNP-46*	BAH84761	alpha/beta hydrolase-fold family protein	Leaf senescence (Morita et al., 2009)
*SNP-47*	GIGAN_ORYSJ	Protein GIGANTEA	Photoperiodic flowering (Fowler et al., 1999)
*SNP-53*	AAT1_ARATH	Transaminase A	Nitrogen assimilation (Schultz et al., 1998)

*Chromosome represents chromosome in which SNP is located; LOCUS represents protein ID in NCBI; Encoded protein represents encoded protein of gene in which SNP is located; Related trait/pathway represents trait/pathway associated with protein*.

## Discussion

### An effective research method to develop markers and analyse the genetic diversity of the wild relatives of wheat

Although the potential of using wild relatives to improve wheat has been recognized for a long time, the available genetic diversity remains largely underexploited. To utilize the full potential of these genes, it is important to understand the genetic diversity in the wild wheat relatives at a molecular level, increase the number of genome-specific molecular markers and identify the loci underlying the traits of interest (Hajjar and Hodgkin, 2007). Due to the large genome complexity and sequence redundancy, reference genomes are not currently available for most wild relatives of wheat. Transcriptome RNA sequencing is an effective strategy for identifying polymorphisms in transcribed regions of the genome. Tanwar et al. (2017) performed a transcriptome sequencing of two guar varieties to develop genomic resources. Potential SSRs and SNPs were identified and 20 SSRs were validated using wet laboratory analysis. Nigam et al. (2017) reported a transcriptome sequencing of closest of wild relative to *Cajanus cajan*. And unigenes and SSR markers were identified in this study. Huang et al. (2016) used Illumina high-throughput deep transcriptome sequencing to characterize of two agriculturally important *Hemarthria* materials. After assembly, unigenes were obtained and annotated and SNPs and SSRs were identified. Some markers were randomly selected to validate the identified markers. However, the low representation of wild wheat relatives in the SNP design process may limit the utility of these platforms in wheat-alien introgression breeding (Wulff and Moscou, 2014). High-quality reference genome assemblies are available for Chinese Spring wheat (Clavijo et al., 2017), which provides a basis for solving this problem. In this study, an effective research method was used to develop genus-specific markers and explore the genetic diversity of wild wheat relatives compared to wheat. First, *de novo* assembly, gene annotation and discovery of genes related to agronomic traits and biotic and abiotic stresses in *A. cristatum* were performed. The comparative genomics analysis revealed that the highest peak value in the sequence identity distribution is 97.6%, and most genes have an identity that is greater than 95% between the *A. cristatum* transcriptome and wheat (Figure 5). The above-mentioned information suggests that there is a close genetic relationship between *A. cristatum* and wheat, which is consistent with previously reported results (Zhang et al., 2015a). Genes with a low similarity between rye sequences and their closest matches in the Triticum genome have been shown to have a higher probability of being repressed or deleted in the allopolyploid genome (Khalil et al., 2015). Therefore, molecular markers developed based on conserved expressed sequences with a high similarity could be applied in wheat improvement because of their high transferability. Based on the concept of discovering variations and revealing genetic diversity between *A. cristatum* and Fukuho for wheat improvement, transcriptome sequencing data were mapped to a wheat reference sequence with a 5% mismatch parameter for Z559, which ensures that the sequencing reads of most genes can be mapped to the wheat reference genome. Based on the mapped results, the discovery of variants and the development of genus-specific markers were performed smoothly and efficiently. The effective research method used in this study can be applied in other studies to investigate genus-specific markers in other wild relatives of wheat.

### Distant relationship and abundant genetic diversity between *A. cristatum* and wheat

Although the wheat-*A. cristatum* derivatives represent an attractive source of value-added traits (Zhang et al., 2015b; Li et al., 2016), the genetic relationship and variants, which represent the fundamental key to breeding success and provide a basis for breeders to select varieties with a constantly improving yield performance (Bedő and Láng, 2015), of *A. cristatum* compared to wheat at the molecular level remain unknown. This study showed that *A. cristatum* had abundant genetic resources and diversity compared to wheat from the various aspects described below. First, phylogenetic analysis indicated that *A. cristatum* is most distantly related to wheat among Triticeae and also showed a clear division between *A. cristatum* and the other Triticeae species, suggesting the P genome possesses rich genetic variation that can be used for wheat genetic improvement (Figure 4). Second, 3,457 genes associated with agronomic traits and biotic and abiotic stress in *A. cristatum* were identified by searching for their homologs in four different genomes (*A. thaliana*, rice, maize, and wheat) (Table S1), and their homologous gene variations were widely distributed in the wheat genome (Figure 6B). In our previous study, 532 genes were associated with biotic stress, 176 genes were associated with abiotic stress and 89 genes with a large spike phenotype were identified in *A. cristatum* (Zhang et al., 2015a). Uncovering novel functional genes paves the way for the efficient mining of the gene pools of wild relatives to improve wheat. Third, 817,970 SNP markers exhibited extremely high allelic variations in wild *A. cristatum* compared to wheat. The SNP density in the wheat D genome was obviously larger than that in the A and B genomes (Figure 7B), indicating that *A. cristatum* is more distantly related to the D genome. This conclusion is also supported by the phylogenetic analysis between P and the wheat A, B, and D genomes (Figure 4). These results were the first to reveal the evolutionary relatedness between *A. cristatum* and the wheat A, B, and D genomes and explained that the wheat A genome and B genome had a higher number of chromosome arrangements with *A. cristatum* chromosomes than the D genome (Badaeva et al., 2007; Li et al., 2016). Interestingly, the variant density in the transcriptional regions was relatively lower in the telomeric and centromeric regions than that in the other chromosomal regions (Figure 6G). This result may explain the phenomenon in which the intercalary translocation lines were difficult to explore in the spontaneous or induced wheat-alien chromosome translocation lines (Jiang et al., 1993), but further research studies are necessary to clarify the molecular mechanisms. Furthermore, the analysis of the evolutionary relationship and genetic diversity of *A. cristatum* compared with wheat, including gene pools related to agronomic traits and biotic and abiotic stress, abundant genetic variation and specific expression genes, provided a good basis for the utilization of *A. cristatum* genomic target regions for the restoration of genetic diversity in future wheat-breeding efforts.

### Future applications of SNP markers

Many types of molecular markers were used in wheat-alien introgression breeding to identify and characterize alien chromosome/chromosome-arm additions and substitution lines. SNP markers provide another means of assessing genetic variation and are abundant and easily obtained via high-throughput sequencing (Vatanparast et al., 2016). Consequently, as a genotyping SNP polymorphism technology, KASP SNP markers are becoming popular and are used in large-scale projects (He et al., 2014; Semagn et al., 2014). Recently, more and more studies have used SNP molecular markers to study alien gene transfer in wheat and detect wheat/wild relative introgressions when they occur (Tiwari et al., 2014, 2015; Winfield et al., 2016; King et al., 2017). In our study, the highest throughput variants, including 817,970 SNPs, between *A. cristatum* and wheat using transcriptome sequencing were identified. Additionally, 37 of the 53 SNPs (69.8%) identified in this study have been verified using KASP genotyping SNP technology. SNP markers developed from transcriptome sequencing are suitable for the development of functional markers that are tightly linked to traits of interest, the large-scale screening of progenies of wild hybrids and the production of lines with introgressed genes of interest and minimum unwanted chromatin (Rey et al., 2015). In addition, SNP markers discovered through transcriptome sequencing are currently used to design genotyping arrays for several important crop plants containing thousands of markers spread throughout the genome to analyse large numbers of samples (Ganal et al., 2012; Sim et al., 2012; Houston et al., 2014; Humble et al., 2016). Therefore, an array using the SNPs discovered in this study can also be developed to detect alien genetic transfer in wheat-*A. cristatum* derivative lines and simplify wheat-alien introgression breeding. Overall, the development of SNP molecular markers in this study can increasingly contribute to incorporating the value-added trait genes from wild relatives to wheat in breeding programmes.

## Author contributions

SZ, LL, and XL designed the study. SZ conducted the bioinformatics analyses and wrote the manuscript. BY and FL performed the experiments. JpZ, JZ, XqL, and XY prepared the samples for sequencing. HM, YL, and WL contributed to editing the manuscript. All authors read and approved the final manuscript.

### Conflict of interest statement

The authors declare that the research was conducted in the absence of any commercial or financial relationships that could be construed as a potential conflict of interest.

## Supplementary material

Table S1 Statistical results of the functionally characterized genes downloaded from GenBank and identified in *A. cristatum*.

Table S2 List of functional genes downloaded from GenBank.

Table S3 A list of effects, with some explanations and putative impacts of the variants.

Table S4 Primer sequences and chromosomes of SNP markers for validation by KASP assay.

Table S5 The distribution of the discovered variants in various genomic features.

File S1 Protein sequences of functional genes downloaded from GenBank (FASTA format).

File S2 Detailed information on functional genes downloaded from GenBank (GenBank format).
